# Selenium Status Is Not Associated with Cognitive Performance: A Cross-Sectional Study in 154 Older Australian Adults

**DOI:** 10.3390/nu10121847

**Published:** 2018-12-01

**Authors:** Barbara R. Cardoso, Ewa A. Szymlek-Gay, Blaine R. Roberts, Melissa Formica, Jenny Gianoudis, Stella O’Connell, Caryl A. Nowson, Robin M. Daly

**Affiliations:** 1 Institute for Physical Activity and Nutrition (IPAN), School of Exercise and Nutrition Sciences, Deakin University, 3220 Geelong, Australia; ewa.szymlekgay@deakin.edu.au (E.A.S.-G.); mprosser@deakin.edu.au (M.F.); j.gianoudis@deakin.edu.au (J.G.); stella.oconnell@deakin.edu.au (S.O.); caryl.nowson@deakin.edu.au (C.A.N.); robin.daly@deakin.edu.au (R.M.D.); 2 The Florey Institute of Neuroscience and Mental Health, Melbourne Dementia Research Centre, Parkville, 3050 Victoria, Australia; blaine.roberts@florey.edu.au

**Keywords:** selenium, cognition, dementia, inflammatory markers, neurotrophic factors

## Abstract

Selenium was suggested to play a role in modulating cognitive performance and dementia risk. Thus, this study aimed to investigate the association between selenium status and cognitive performance, as well as inflammatory and neurotrophic markers in healthy older adults. This cross-sectional study included 154 older adults (≥60 years) from Victoria, Australia. Participants were assessed for cognitive performance (Cogstate battery), dietary selenium intake (two 24-h food recalls), plasma selenium concentration, inflammatory markers (interleukin (IL)-6, -8, -10, tumor necrosis factor-alpha and adiponectin) and neurotrophic factors (brain-derived neurotrophic factor, vascular endothelial growth factor and insulin-like growth factor 1). Dietary selenium intake was adequate for 85% of all participants. The prevalence of selenium deficiency was low; only 8.4% did not have the minimum concentration in plasma required for optimization of iodothyronine 5′ deiodinases activity. Multiple linear regression analysis revealed that plasma selenium was not associated with cognitive performance, inflammatory markers nor neurotrophic factors, independent of age, sex, body mass index (BMI), habitual physical activity, *APOE* status, education, and history of cardiovascular disease. The lack of association might be due to the optimization of selenoproteins synthesis as a result of adequate selenium intake. Future prospective studies are recommended to explore potential associations of selenium status with age-associated cognitive decline.

## 1. Introduction

Ageing is accompanied by structural and functional brain deterioration, as well as a decline in cognition. The cognitive decline may progress to mild cognitive impairment (MCI) and dementia, affecting the capacity of daily living activities and reducing quality of life [1].

Dementia is one of the major causes of disability and dependency among older people, and is one of the top ten leading causes of death globally [2]. Since there is no cure for dementia, there has been considerable interest in identifying and addressing modifiable risk factors for early cognitive changes in non-demented older people. It has been projected that controlling seven of the most relevant modifiable risk factors, including mid-life obesity, physical inactivity, smoking, low educational attainment, depression, diabetes mellitus, and hypertension, would delay the onset of dementia and reduce its incidence by up to 30% [3,4]. However, there is also emerging research indicating that various nutrients, dietary habits, and/or patterns may play an important role in modulating cognitive performance [5,6].

Among different dietary compounds that have been assessed in relation to cognitive function and dementia risk, the essential micronutrient selenium may be of particular relevance due to its importance for the maintenance of brain homeostasis [7]. The biological effects of selenium on brain health are mediated by selenium-containing proteins (selenoproteins), with evidence that this micronutrient is utilised by cells via the presence of selenocysteine (SeCys) amino acid residues in 25 identified selenoproteins. Among the different roles played by selenoproteins, it is noted that at least one third have antioxidant characteristics, which is essential for neuroprotection [7]. Furthermore, in vitro and animal studies have demonstrated that selenium is associated with increased level of brain-derived neurotrophic factor (BDNF) [8], regulation of Ca^2+^ channels and mitochondrial biogenesis in response to stress and modulating apoptotic pathways in neuronal cells [9,10,11,12]. More recently, the selenoprotein glutathione peroxidase 4 was identified as a key regulator of ferroptosis, a newly identified form of programmed cell death dependent on iron that causes a rapid elevation in the levels of reactive oxygen species [13,14]. Furthermore, the brain ranks atop on the selenium hierarchy, as its stores are mostly preserved in cases of deficiency and it is the first organ to return to normal levels when selenium exposure becomes replete, strengthening the importance of selenium for the homeostasis of the central nervous system [15].

Selenium is also negatively associated with markers of systemic inflammation, which has a pivotal role in age-associated cognitive decline and dementia pathogenesis [16,17,18]. For instance, in vitro studies have demonstrated that low selenium concentration up-regulates pro-inflammatory genes via activation of nuclear factor kappa B (NFkB), while increased selenium supply reduces activation of this pathway in several cells, such as neurons [19], macrophages [20], prostate [21], and gut epithelium [22]. Corroborating these findings, improvement of selenium status with Brazil nut intake was associated with a decreased serum interleukin (IL)-6 and tumor necrosis factor (TNF)-alpha response in hemodialysis patients via modulation of nuclear factor kappa B (NFkB) pathway and increased expression of nuclear factor E2-related factor 2 (*NRF2*) and NAD(P)H: quinone oxidoreductase 1 (*NQO1*) genes [23,24]. Collectively, these findings suggest a potential anti-inflammatory effect of selenium that may positively increase brain resilience against age-associated brain pathologies.

Human studies have suggested that decreased selenium status is associated with Alzheimer’s disease pathogenesis, as individuals with this disease present with lower selenium concentration in different biomarkers, such as blood, cerebrospinal fluid and nails, when compared with healthy controls [25,26,27]. This hypothesis is corroborated by several studies that reported a positive association between selenium status and cognitive performance in older adults [25,28], suggesting that optimizing selenium may play a role in reducing the risk of age-associated cognitive decline. However, these studies were conducted in areas where selenium intake was low due to poor selenium content in locally sourced food, and thus selenium deficiency was highly prevalent. Previous research in Australia has shown that selenium intake and status are typically higher than that reported in other countries [29,30], but it remains unclear whether selenium status is associated with cognition in relatively healthy older Australian adults. Hence, the aim of this study was to investigate the association between selenium status and cognitive performance, as well as inflammatory and neurotrophic markers in older Australian adults.

## 2. Materials and Methods

### 2.1. Study Design

This cross-sectional study used data from the Seniors’ Thinking, Exercise and Protein Study (STEPS), which was a randomised controlled trial that aimed to assess the effects of an exercise program combined with a protein-enriched diet on cognitive function, skeletal muscle mass, size, and strength in older Australian adults [31].

This study was conducted according to the guidelines laid down in the Declaration of Helsinki and all procedures involving human subjects were approved by the Deakin University Human Research Ethics Committee (DUHREC), Burwood, Victoria, Australia (protocol number 2013-166). Written informed consent was obtained from all subjects prior to their inclusion in the study. The trial was registered at the Australian New Zealand Clinical Trials Registry (www.anzctr.org.au): ACTRN12613001153707.

### 2.2. Participants

A total of 154 community dwelling elderly aged ≥60 years were recruited from metropolitan Melbourne and surrounding areas in Victoria, Australia. Participants were initially screened over the telephone and excluded on the following criteria: current or prior participation (past 3 months) in ≥150 min per week of moderate intensity physical activity or resistance exercise >1 day per week; acute or terminal illness such as cancer and related treatment; recent low trauma fracture; use of insulin to treat diabetes; self-report height and weight that resulted in a body mass index (BMI) >40; coeliac disease; ulcerative colitis or inflammatory bowel disease; Crohn’s disease; chronic liver disease; or recent use of oral corticosteroids (past six months). Eligible participants were then screened for depression and cognitive performance with the Geriatric Depression Scale (GDS) [32] and the Short Portable Mental Status Questionnaire (SPMSQ) [33], and those with a score of >6 on the GDS or a score of >2 on the SPMSQ were not included in the trial. Finally, participants were asked to provide a fasted, morning blood sample to confirm that their estimated Glomerular Filtration Rate (eGFR) was >45 mL/min/1.73 m^2^. The final recruited sample comprised of 154 individuals aged 70.7 (4.1) years. Written informed consent was obtained from all participants, and the study was approved by the Deakin University Human Research Ethics Committee (HREC 2013–166).

### 2.3. Health and Demographics

A socio-demographic and lifestyle questionnaire was used to obtain information on education background and history of disease(s). Education was categorized as completion of primary/high school, technical certificate, or university. History of cardiovascular disease (CVD) was defined as self-reported history/presence of heart disease, stroke or heart attack.

### 2.4. Biological Samples

A fasted morning (8–10 a.m.) venous blood sample was collected at a commercial pathology clinic with multiple collection centers in Melbourne, Victoria, Australia. Samples were sent to a central laboratory for processing. Plasma or serum was separated by centrifugation at 3000 *g* for 15 min at 4 °C and stored at −80 °C until analysis for selenium and the inflammatory and neurotrophic markers.

### 2.5. Plasma Selenium

Selenium concentration in plasma was measured using inductively coupled plasma-mass spectrometry (ICP-MS), according to a previously described method [34]. Briefly, neat plasma was diluted in 1% nitric acid (1:20 to 1000 µL final volume). Selenium was measured on mass at *m*/*z* = 78 (^78^Se; natural abundance = 23.8%) using an Agilent Technologies 7700x ICP-MS system (Agilent Technologies, Melbourne, Australia) fitted with “cs” lenses and platinum cones. Hydrogen (4 mL min^−1^) was used as a reaction gas to remove polyatomic interferences at *m*/*z* = 78. An external calibration curve was prepared using a multi-element standards solution (AccuStandard, New Haven, CT, USA) containing 0, 0.1, 0.5, 1, 5, 10, 50, and 100 µg L^−1^ of selenium. An internal standard solution containing 200 µg L^−1^ of yttrium (^89^Y) was introduced online via a Teflon T-piece. Analytical validity was assessed using reconstituted lyophilized Seronorm™ Trace Elements in Serum (Sero AS, Billingstad, Norway) standard reference material, which was prepared using the same protocol as for plasma samples. The measured analytical recovery of selenium in the Seronorm™ standard was within the acceptable range, per manufacturer’s guidelines [measured serum = 122.18 (6.56) µg L^−1^; certified range = 95–176 µg L^−1^; *n* = 10].

### 2.6. Inflammatory and Neurotrophic Markers

Inflammatory and neurotrophic markers were measured in serum. High sensitivity C-reactive protein (hs-CRP) was measured by an immunoturbidimetric assay from Roche Diagnostics, (Roche Diagnostics, Mannheim, Germany). Interleukines (IL)-6 (IL-6), IL-8, IL-10 and tumor necrosis factor-alpha (TNF-alpha) were measured using the Milliplex T Cell high-sensitivity human cytokine panel (Millipore, Billerica, MA, USA) as per manufacturer’s recommendations with an intra-assay coefficient of variability (%CV) of 5.9–11.7% and an inter-assay %CV of 7.3–15.7%. Adiponectin was assessed using Procarta kit (Affymetrix, Fremont, CA, USA) as per manufacturer’s guidelines with an intra-assay %CV of 6.8% and an inter-assay %CV of 8.4%. Each inflammatory marker was converted into a *z*-score and a single composite inflammatory score was created as follows: sum of all pro-inflammatory markers (hs-CRP, IL-6, IL-8 and TNF-alpha) minus the sum of all anti-inflammatory markers (IL-10 and adiponectin). A higher *z*-score is more representative of a pro-inflammatory status.

Serum brain-derived neurotrophic factor (BDNF) and vascular endothelial growth factor (VEGF) were determined by ELISA using the commercial Duo kit ELISA (R & D Systems, Minnneapolis, MN, USA) as per manufacturer’s guidelines with an intra assay %CV of 3.9–5.9% and an inter-assay %CV of 4.4–14.7%. Insulin-like growth factor 1 (IGF-1) was measured using the Immulite 2000 IGF-1 chemiluminescent immunometric assay (Siemens Healthcare Diagnostics, Los Angeles, CA, USA), with an intra-assay %CV of 3.1 and inter-assay %CV of 6.2.

### 2.7. Cognitive Function

The Cogstate computerised battery was administered to assess the domains of attention, processing speed, memory and executive function [35]. This battery has been validated in older adults, with minimal practice or fatigue effects, and provides highly sensitive information to detect cognitive impairment. Participants were asked to complete five tasks on a laptop with a mouse and headphones provided. The tasks were as follows: (i) Detection task (DET), that measures speed of processing and psychomotor function; (ii) Identification task (IDN), that measures visual attention; (iii) One card learning task (OCL), that assesses visual recognition memory and attention; (iv) One-back task (ONB), that measures working memory and attention; and (v) Groton maze learning task (GML), that measures executive function, memory and spatial problem solving. DET, IDN, and ONB were scored using speed (reaction time in milliseconds); OCL was scored using the number of correct responses made (accuracy); and the GML task was scored using the total number of errors on five consecutive trials at a single session [36,37]. Raw scores were transformed into a *z*-score using the mean and standard deviation of the total sample in the study. From the above five tests, three composite scores were computed [35]: (i) Working memory/learning: computed by averaging speed *z*-scores for OCL and ONB; (ii) Attention/psychomotor function: computed by averaging the *z*-scores for DET and IDN; (iii) Global cognitive function: computed by averaging the *z*-scores for all five tasks.

Participants also completed the Behaviour Rating of Inventory of Executive Function-Adult Version (BRIEF-A), a self-report questionnaire of executive function. The 75 questions make up nine non-overlapping theoretically and empirically derived clinical scales that provide a measure of various aspects of executive functioning, such as inhibit, self-monitor, plan/organise, shift, initiate, task monitor, emotional control, working memory, and organisation of materials [38]. The scores from inhibit, shift and emotional control were summed together to provide the Behavioural regulation index score, and the other subdomains were summed to provide the Metacognition index score. Behavioural regulation and Metacognition indices were then summed together to provide the Global executive composite. T-scores were derived for each scale, with higher scores representing a greater degree of executive dysfunction.

### 2.8. Physical Activity

Total physical activity was assessed using the Community Healthy Activities Model Program for Seniors (CHAMPS) physical activity questionnaire developed and validated for use with older adults [39]. Results were reported as estimated kilojoules per week spent in moderate-to-vigorous intensity activities.

### 2.9. Anthropometry

Body mass index (BMI, kg m^−2^) was derived from height, measured to the nearest 0.1 cm with a wall-mounted stadiometer, and bodyweight, measured to the nearest 0.1 kg using calibrated electronic digital scales. Normal weight was categorized as 18.5–24.9 kg m^−2^, and overweight and obesity were categorized as 25.0–29.9 and ≥30 kg m^−2^ [40].

### 2.10. Selenium Intake

Dietary intake was assessed from two 24-h food recalls. Selenium intake was analysed using Australia-specific dietary analysis software (Foodworks 7, Xyris Software, Highgate Hill, Australia). Inadequacy of selenium intake was estimated as below 60 µg day^−1^ for men and 50 µg day^−1^ for women, according to the Estimated Average Requirement (EAR) established by Nutrient Reference Values for Australia and New Zealand [41].

### 2.11. APOE Genotype

*APOE* genotype, the strongest genetic risk factor for Alzheimer’s disease with effect on memory over the adult life course [42], was assessed through a venous blood sample. Genetic determination of *APOE* allelic status was performed using a polymerase chain reaction (PCR)-based assay designed with the MassARRAY Assay Design 4.0 software (Agena Bioscience, San Diego, CA, USA). The initial PCR step involved 45 cycles with an annealing temperature of 56 °C. The PCR products were treated with shrimp alkaline phosphatase for 15 min at 37 °C and denatured at 85 °C for 5 min. The final iPLEX extension step involved 40 cycles of lots of five cycles between 52 °C and 85 °C. The resulting iPLEX extension products were desalted using SpectroCLEAN resin (SEQUENOM, San Diego, CA, USA), then spotted on SpectroCHIPs GenII (SEQUENOM, San Diego, CA, USA) and analysed with the MassARRAY Analyser Compact MALDI-TOF MS (Agena Bioscience, San Diego, CA, USA). Participants were categorised as possessing at least one copy of the ε4 allele from the *APOE* (ε4 carrier) or no copies of this polymorphism (non-ε4).

### 2.12. Statistical Analysis

All statistical analyses were performed using the Statistical Package for the Social Sciences software (v 24.0) for Windows (IBM, Armonk, NY, USA). Data distribution for all measures was verified with Kolmogorov–Smirnov test and the homogeneity of variance with Levene test. The neurotrophic markers (IGF-1, BDNF, VEGF) were log transformed prior to analysis. Multiple linear regression models were used to examine the relationship between plasma selenium concentrations and cognitive outcomes. All models were adjusted for age, sex, BMI, habitual physical activity, genotypes for *APOE* (carriers of ɛ4/non-carriers), education level, and history of CVD. Linear regression models were also used to examine the association between plasma selenium concentration and the composite inflammatory index and neurotrophic markers (IGF-1, BDNF, and VEGF). These models were adjusted for the same covariates, except for education. Pearson’s correlation was used to assess the association between dietary and plasma selenium. A *p* value < 0.05 was considered significant.

## 3. Results

The characteristics of the 154 participants included in this study are shown in Table 1. On average, the participants were aged 70.7 years (range 64.6 to 83.6 years), with the majority being female (62%), one-third (32%) classified as obese, and 21% as carriers of *APOE*ɛ4. Most participants were cognitively healthy, as only seven were classified as having cognitive impairment (MCI) based on a z-score of ≤ −1.0 SD on at least three of the five individual cognitive tests from the CogState battery [35]. There were no differences in the characteristics of participants with and without MCI, with the exception that BMI was significantly lower for those with MCI [22.4 (3.2) and 28.1 (5.2) kg m^−2^ respectively, *p* = 0.005].

**Figure 1:**
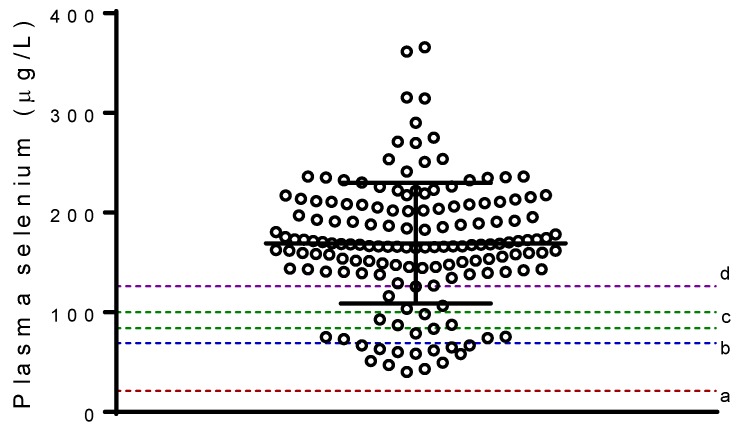
Plasma selenium concentration. Horizontal line corresponds to mean, and error bars to standard deviation. Dashed lines correspond to selenium concentration necessary for: (a) prevention of Keshan disease (>21 µg L^−1^), (b) optimal activity of iodothyronine 5′ deiodinases (IDIs) (>69 µg L^−1^), (c) maximization of plasma GPx activity (84–100 µg L^−1^), (d) protection against some cancers (>126 µg L^−1^).

Dietary selenium intake was adequate for most of the participants, as 85% of them met the Australian EAR. Selenium intake was positively correlated with plasma selenium (*r* = 0.253, *p* = 0.002). The prevalence of selenium deficiency based on the plasma samples was low in this study cohort. No participants presented with a risk for Keshan disease (<21 µg L^−1^); only 8.4% (*n* = 13) did not have the minimum plasma selenium concentration required for optimization of iodothyronine 5′ deiodinases (IDIs) activity (>69 µg L^−1^); 12.3% (*n* = 19) did not have adequate concentration for optimization of glutathione peroxidase (GPx) (84–100 µg L^−1^) [43]; and 81.2% presented with a plasma selenium concentration above the cut-off associated with significant decrease in risk for cancer (>126 µg L^−1^) [43] (Figure 1).

Multiple linear regression analysis revealed that plasma selenium status was not associated with any of the cognitive outcomes (Table 2). Similarly, plasma selenium concentration was neither associated with the composite inflammatory index (or any of the individual inflammatory markers—data not shown) nor any neurotrophic markers (Table 3).

## 4. Discussion

The main finding from this study was that plasma selenium status was not associated with cognitive performance in this cohort of relatively healthy Australian older adults who mostly had adequate dietary selenium intakes and plasma selenium concentrations. In addition, there was no association between plasma selenium and any circulating inflammatory or neurotrophic markers.

The finding that plasma selenium concentration was adequate in our cohort of Australian older adults is likely due to the high exposure through diet, as demonstrated by the correlation between dietary selenium intake and plasma selenium concentrations. This is in agreement with other studies conducted in Australia that reported a low prevalence of inadequate selenium intakes [29,30,44], and with the observations from a previous study we conducted with participants from Melbourne and Perth [34]. In this previous study, only one individual (1.1%) presented with insufficient plasma selenium for glutathione peroxidase optimization. A high selenium content in foods grown in Australia was reported by Fardy et al. [45] when assessing selenium concentration in 50 representative foods from each of the seven Australian state capitals. Combining these data with hypothetical diets, they estimated that daily selenium intake for Australian men and women was 87 mg day^−1^ and 57 mg day^−1^, respectively [45], which are above the EAR (60 µg day^−1^ for men and 50 µg day^−1^ for women) that is associated with optimization of selenoprotein synthesis [46].

The finding that the serum selenium status was not related to cognitive function in our study appears to contrast with the findings from several previous studies that assessed selenium and cognition in community-dwelling older adults [25,28,47]. These contrasting results are likely to be due, at least in part, to differences in the selenium status of participants. In our study of healthy older Australian adults, the average serum selenium concentration was 170 µg L^−1^, whereas in several previous studies reporting an association between cognition and selenium status, selenium concentration was markedly lower [25,28,47]. For instance, in a prospective study of 1166 high cognitively functioning French adults aged 60–70 years, lower plasma selenium status (mean 87 (SD 16) µg L^−1^) was associated with subsequent cognitive deterioration over 4 years of follow-up [28]. Similarly, a study of 1012 Italian adults aged 65 years or older with a mean plasma selenium concentration of 74.5 µg L^−1^ reported that there was a trend for a positive association between plasma selenium and cognitive performance [47]. Another cross-sectional study conducted with Chinese elderly people showed that decreased selenium concentration in nail samples were associated with lower cognitive scores [25]. In that study, dietary selenium intakes were extremely low, ranging from 9.75 to 46.73 µg day^−1^, which contrasts with the results from our population.

The impact of different selenium concentrations (or status) also seems to result in conflicting data when evaluating the results from clinical trials. For instance, findings from the PREADVISE study revealed no effect of selenium supplementation on Alzheimer’s disease risk in male older adults with adequate selenium status [48]. In contrast, increased selenium supply for six months with one Brazil nut (c.a. 288.8 µg selenium/day) was demonstrated to improve verbal fluency and constructional praxis in mildly cognitively impaired older adults with selenium deficiency [49]. While differences in plasma selenium concentrations between studies may reflect different assay methods, collectively these studies provide some evidence that low plasma selenium concentrations are associated with poorer cognitive performance in older adults. On the other hand, adequate selenium as observed in our study has been associated with maximization of selenoprotein synthesis [43,50]. As a consequence, the plateaued expression of selenoproteins may explain the lack of association between selenium status and cognition performance in our study. Nevertheless, it would be worth following up the participants of this study over time to explore whether there might be a potential association with selenium status and age-associated cognitive decline.

It is well established that ageing is associated with structural and functional brain changes that lead to age-related cognitive decline, including a reduction in brain volume, synaptic density and plasticity, and an increase in oxidative stress and inflammation [1,51], and that selenium has been demonstrated to protect neurons against these insults [7,52]. Similarly, neurotrophic factors are also associated with maintenance of neuronal plasticity and survival, and thus are considered essential factors for brain resilience [53]. In our study, selenium was not associated with any neurotrophic factors, and we speculate that the lack of any such association was because synthesis of selenoproteins had reached the highest plateau due to a high dietary selenium intake. This may also explain why we observed no association between selenium and any inflammatory markers in our cohort. This hypothesis is in alignment with the assumption that there is no rationale for giving selenium supplements to cognitively healthy population groups with sufficient selenium intake and adequate status [54].

A strength of this study is the inclusion of multiple markers for neurotrophic and inflammatory factors to provide an index of inflammatory status along with cognitive outcomes, as these biomarkers are not usually assessed in studies that investigate association between selenium status and cognition. However, there are a number of limitations. Our study included only 154 older adults that presented sufficient selenium status, and thus this limits the generalizability of the findings to all older adults. In addition, this was a cross-sectional study which limits causality and sample size or power calculations were not performed on the outcomes reported in this study as this was a secondary analysis from a larger intervention trial. Furthermore, we assessed only one short-term selenium biomarker, which precludes conclusions associated with long-term selenium status. Regarding inflammatory and neurotrophic markers, although blood circulating concentrations provide information about their periphery roles, it is known that cerebrospinal fluid better represents the main export pathway from the central nervous system. However, blood markers present the advantage of being less invasive, more acceptable to patients, and cost and time-effective [55].

## 5. Conclusions

Selenium concentration was not associated with cognitive performance or with inflammatory or neurotrophic factors in selenium-replete older adults. The lack of association might be due the optimization of selenoproteins synthesis as a result of adequate selenium intake in the study population. These results should be further interpreted having into account the cross-sectional design and the small sample size of the study. Future prospective studies would be warranted to explore potential associations of selenium status with age-associated cognitive decline.

## Figures and Tables

**Table 1:** Population characteristics (*n* = 154).

Parameter	Values
Age (year)	70.7 (4.1)
Women, *n* (%) ^a^	96 (62.3)
*APOE*ɛ4 carriers, *n* (%) ^a^	33 (21.4)
BMI (kg m^2^)	27.9 (5.3)
Overweight, *n* (%) ^a^	54 (35.1)
Obese, *n* (%) ^a^	49 (31.8)
Education	
Primary/high school, *n* (%) ^a^	57 (37)
Technical certificate, *n* (%) ^a^	25 (16.2)
University, *n* (%) ^a^	72 (46.8)
Habitual physical activity (kJ week^−1^)	7957.3 (7526.5)
History of CVD, *n* (%) ^a^	23 (14.9)
Selenium intake (µg day^−1^)	93.1 (53.6)
Plasma selenium (µg L^−1^)	169.3 (60.4)
Serum IGF-1 (nmol L^−1^)	17.1 (4.7)
Serum BDNF (ng mL^−1^)	21.3 (7.7)
Serum VEGF (pg mL^−1^)	407.5 (455.1)
Serum hs-CRP (mg/mL)	2.1 (2.6)
Serum IL-6 (pg/mL)	4.6 (7.9)
Serum IL-8 (pg/mL)	15.6 (10.5)
TNF-alpha (pg/mL)	10.2 (3.9)
IL-10 (pg/mL)	10.7 (17.1)
Adiponectin (µg/mL)	5.1 (3.5)
Composite inflammatory index ^b^	0.00 (2.4)

Values represent mean (SD) unless stated; ^a^ Number (proportion); ^b^ Composite inflammatory index calculated as sum of individual *z*-scores of pro-inflammatory markers (hs-CRP, IL-6, IL-8 and TNF-alpha) minus anti-inflammatory markers (IL-10 and adiponectin). APOE, apolipoprotein E; BDNF, brain-derived neurotrophic factor; BMI, body mass index; CVD, cardiovascular disease; IGF-1, insulin-like growth factor 1; VEGF, vascular endothelial growth factor.

**Table 2:** Associations between plasma selenium concentration and cognitive outcomes in 154 older adults.

Outcomes	β	95% CI	*p*
Cogstate			
Global cognitive function composite ^a^	0.001	−0.001; 0.003	0.198
Working memory/learning composite ^a^	0.002	0.000; 0.004	0.095
Attention/psychomotor composite ^a^	0.002	−0.001; 0.004	0.149
BRIEF-A			
Behavioural regulation index ^b^	−0.016	−0.042; 0.010	0.214
Metacognition index ^b^	−0.013	−0.041; 0.015	0.369
Global executive composite ^b^	−0.015	−0.041; 0.011	0.255

^a^ Cogstate, *z*-score; ^b^ Behaviour Rating of Inventory of Executive Function-Adult Version (BRIEF-A), *t*-score; β represents unstandardized beta-coefficients. Results were adjusted for age, sex, BMI, habitual physical activity (kJ weekly spent in vigorous activity), genotypes for *APOE* (carriers of ɛ4/non-carriers), education (primary/high school/technical certificate/university), and history of cardiovascular disease (CVD) (yes/no).

**Table 3:** Associations between plasma selenium concentration and the composite inflammatory index and neurotrophic markers in 154 older adults.

Outcomes	β	95% CI	*p*
Composite inflammatory index ^a^	0.002	−0.005; 0.009	0.550
IGF-1 (nmol L^−1^) ^b^	0.000	−0.001; 0.000	0.238
BDNF (ng mL^−1^) ^b^	0.000	−0.001; 0.001	0.799
VEGF (pg mL^−1^) ^b^	−0.001	−0.003; 0.002	0.687

^a^*z*-score; ^a^ Composite inflammatory index calculated as sum of individual *z*-scores of pro-inflammatory markers (hs-CRP, IL-6, IL-8 and TNF-alpha) minus anti-inflammatory markers (IL-10 and adiponectin); ^b^ log transformed values; β represent unstandardized beta-coefficients. Results were adjusted for age, sex, BMI, habitual physical activity (kJ weekly spent in vigorous activity), genotypes for *APOE* (carriers of ɛ4/non-carriers) and history of CVD (yes/no).
